# Vertebrate behavioral thermoregulation: knowledge and future directions

**DOI:** 10.1117/1.NPh.11.3.033409

**Published:** 2024-05-20

**Authors:** Bradley Cutler, Martin Haesemeyer

**Affiliations:** a Graduate program in Molecular, Cellular and Developmental Biology, Columbus, Ohio, United States; b The Ohio State University, Columbus, Ohio, United States

**Keywords:** thermoregulation, homeostasis, circuits, computation, behavior, imaging

## Abstract

Thermoregulation is critical for survival across species. In animals, the nervous system detects external and internal temperatures, integrates this information with internal states, and ultimately forms a decision on appropriate thermoregulatory actions. Recent work has identified critical molecules and sensory and motor pathways controlling thermoregulation. However, especially with regard to behavioral thermoregulation, many open questions remain. Here, we aim to both summarize the current state of research, the “knowledge,” as well as what in our mind is still largely missing, the “future directions.” Given the host of circuit entry points that have been discovered, we specifically see that the time is ripe for a neuro-computational perspective on thermoregulation. Such a perspective is largely lacking but is increasingly fueled and made possible by the development of advanced tools and modeling strategies.

## 1 Introduction

Temperature sensation is an ancient process that is critical for survival across organisms. The biomolecules that cells require to function have an optimal temperature at which they operate. Therefore, organisms that can detect and seek preferable temperatures can better adapt to their environment.^1^^,^^2^ This has led to the evolution of intricate thermoregulatory control systems across bacteria,^3^^–^^5^ plants,^6^ and invertebrate and vertebrate animals.^7^^,^^8^ Accordingly, there are different proteins, ribonucleic acids (RNAs), and even membrane lipids that serve as molecular thermometers for many lifeforms and cell types.^9^^,^^10^ We will focus this review on vertebrates with an emphasis on behavioral thermoregulation. Work over the past decades has led to an in-depth understanding of thermosensory pathways and thermoregulatory effector systems, especially in mammals.

Here, we will review knowledge on the cellular–molecular basis of thermoregulation, the afferent pathways, and how peripheral thermosensory information is integrated with information about body temperature and internal states. We will end the review with what we believe to be the next set of important questions that are all related to the need of understanding thermoregulation as a dynamic process: Knowledge of how neurons across brain regions orchestrate thermoregulatory behavior is largely lacking. We believe that this fundamentally limits our understanding of how the brain controls body temperature, necessitating new complementary approaches to the study of thermoregulation.

## 2 Main Text

### 2.1 Molecular Thermometers and Their Organization in Neural Thermosensation

To thermoregulate, the nervous system must first sense temperature by converting environmental thermodynamic information into membrane potential changes. Transient receptor potential (TRP) channels form the best-studied part of the molecular machinery that converts temperature into neural activity.^11^^,^^12^ TRP channels are elegantly arranged proteins that undergo conformational change to allow cations to enter neurons under specific temperature conditions tying neural depolarization to thermal stimuli.^13^

The TRP sensors are a family of nonspecific cation channels that act as signal transduction conduits, translating environmental conditions such as heat or mechanical force into membrane potential. Here, we will present a whirlwind tour of thermosensitive TRP channels and their functional motifs to provide a mechanistic overview of this molecular thermometer superfamily. Many TRP family proteins contain binding sites that can be used by ligands to activate or modulate the channel’s activity.^11^ From phosphatidylinositols to hormones and peptides, TRP channels are sensitive to a wide range of molecules that act as signal transducers for biological signaling cascades in addition to environmental conditions.^14^^–^^16^

Caterina et al.^17^ found that the capsaicin receptor TRPV1 responds to noxious heat in addition to the spicy molecule in neurons. The TRPV family is named after their ability to bind to vanilloid compounds. Capsaicin is a vanilloid that acts as a strong agonist to TRPV1 evoking a sensation of heat in downstream circuits.^17^^–^^21^ Importantly, the sensation is tied to where the channel is expressed and how neurons expressing it are wired. Capsaicin expression was co-opted by plants to evoke painful heat sensations that would reduce predation in a species-specific manner.^22^

Clever experiments using molecular agonists were successful in describing the structure of TRPV1 in various states of activation.^13^ However, there are obvious challenges to detecting different conformations of a heat-activated channel with crystallography where crystals tend to preferentially form at specific temperatures or cryo-EM, which relies on freezing samples.^23^ However, in 2021, Kwon et al.^24^ used a combination of agonists and lipid nanodisc technology to capture the closed, a few intermediate, and fully open state of TRPV1 at temperatures below its heat-dependent activation temperature of 42°C using capsaicin variants as an agonist. They hypothesized that a series of heat capacity changes across the tertiary structure of the protein results in a conformational cascade causing hydrophobic residues to retract from the pore, ultimately allowing for cations to pass through.^24^^–^^26^ The structural motifs permitting the thermosensitivity of individual TRP channels vary. However, there is a common theme involving the sum of minor structural changes driven by denaturation events that move amino acids within the path ions traverse through the channel. One could argue that findings on TRPV1 using lipid nanodiscs do not have a direct link with temperature, and the changes observed are due to agonist binding and do not represent an exclusive heat-sensitivity mechanism. However, creative cryo-EM techniques using TRPV3 were in line with the TRPV1 findings. Here, the authors captured the open, closed, and intermediate conformations driven by temperature, showing that accumulated denaturation events are responsible for temperature-driven TRPV3 channel opening as well.^27^

The canonical cold receptor is TRPM8.^28^^,^^29^ This channel is also gated by the compound menthol, which is therefore perceived as a cooling sensation much like capsaicin signaling through TRPV1 is perceived as hot. TRPM8 exhibits a similar mode of functioning to TRPV1; however, considering that TRPM8 is activated by cold rather than heat, it is less clear if a large series of heat capacity changes (denaturation events) within the molecule leads to major conformational changes. The opening of the TRPM8 inner pore is actuated by a set of hydrophobic amino acids being raised and lowered as a physical gate similar to the mode of opening seen in TRPV channels, suggesting that the mechanisms of hot and cold sensitivity might be comparable.^30^ Heat capacity calculations of TRPM8 provided theoretical proof that many coordinated denaturation events within the internal structure of a protein subunit instigated by a temperature above 28°C could drive downstream conformational changes, closing the pore.^31^ When assessed in this manner, the stated research conceptualizes temperature-responsive TRP channels as thermodynamically balanced proteins that use a domino effect of seemingly insignificant temperature-driven denaturation events within their internal structure to drive large shifts of pore structures and thereby allow or deny cation flow.

Interestingly, TRPM8 appears to be absent from fish genomes,^32^ and it has been suggested that TRPM8 only evolved its cold-sensing domain after the water-to-land transition.^33^ This predicts the existence of alternative means to sense cold in fish, which are currently unknown. Some fish species are highly cold tolerant, thriving in temperatures below 4°C, and these species may therefore survive without the ability to sense cold; however, this does not explain the absence of TRPM8 in tropical species such as zebrafish, which very likely, and maybe secondarily, acquired alternate means to detect cold temperatures.^32^ A similar question arises about warm-sensing in strongly cold-adapted fish. Antarctic fish exclusively survive at temperatures below 4°C, yet they seem to express canonical warm receptors such as TRPV1.^34^ Furthermore, the Antarctic fish isoform of this channel appears to gate only at temperatures above 22°C.^35^ It is therefore unclear whether these fish are able to avoid warmth or if they instead rely on a very stable environmental temperature and lack said ability.

While TRP sensors are a major way for our cells to detect temperature, one must remember that the TRP protein family serves very diverse functions. Specifically, TRP channels are present within many different cell types. This includes immune, digestive, pulmonary, and cardiac cells to name a few.^36^ TRP channels are also involved in signaling cascades independent of any thermoregulatory role. As such, TRP channel signaling can affect diverse signaling pathways, some directly and others indirectly through calcium influx.^37^ These pathways cause modulation of developmental, immune, and healing processes and at the same time may link physiological events to thermosensation.^38^ This functional diversity may explain why not all TRP sensors involved in thermal computations are temperature sensitive, e.g., TRPC4 appears necessary for sensing warm temperatures within the preoptic area of the hypothalamus (POA), but the channel itself is not a temperature sensor.^39^

The research just covered shows that certain TRP sensors serve as a molecular thermometer, utilized by neurons to detect local temperatures in the form of cation flow (Table 1). As a group, TRP channels are thought to tile temperature space such that different temperature-sensitive TRP channels require specific temperatures to allow ion flow.^11^ The TRPV family is largely involved in detecting warm (TRPV3 and TRPV4) as well as hot or noxious temperatures (TRPV1, TRPV2) with opening thresholds ranging from 30°C to 50°C.^57^ In addition, TRPM2 has been shown to be required for the sensation of innocuous warmth.^43^ Importantly, the context of expression of the channel *in vivo* is known to change thermal activation thresholds. While TRPV1 is classified as sensing noxious temperature when analyzed in heterologous systems, it appears to be involved in the detection of innocuous warmth in the mouse trigeminal ganglion (TG).^41^ On the cold side, TRPM8 senses ambient cold between 8°C and 20°C.^42^ While TRPA1 had been proposed as a sensor of noxious cold (a counterpart to TRPV1 and TRPV2), this role is controversial and TRPA1 is rather believed to be an important chemosensor.^46^^,^^54^ Instead, noxious cold is likely sensed by the kainate glutamate receptor GluK2 via a G protein-coupled mechanism.^55^^,^^56^

**Table 1:** Identified temperature sensors and their functions.

Protein	Comments	Selected references
TRPV1	• Generally thought of as a noxious temperature sensor, activating at temperatures above 40°C *in vitro*	17, 18, 24, 35, 40, and 41
	• In the mouse TG, TRPV1 marks neurons responding to innocuous warmth	
	• Antarctic fish and zebrafish TRPV1 activate at lower temperatures of 20°C to 25°C	
	• Sensitive to capsaicin in many species	
TRPM8	• Canonical cold sensor	29, 30, 33, and 42
	• Absent from most fish and acquired cold-sensing domain on the water-to-land transition	
	• Sensitive to menthol	
TRPM2	• Presynaptic warm-sensor in the preoptic area	43 [Bibr r44] – 45
	• Indirectly enhances activity in warm-sensing POA neurons when temperature increases	
TRPC4	• Required for warm sensitivity in preoptic area warm-sensing neurons but not itself a thermosensor	39
TRPA1	• Contested role in the sensation of noxious cold	46 [Bibr r47] [Bibr r48] [Bibr r49] [Bibr r50] [Bibr r51] [Bibr r52] [Bibr r53] – 54
	• Noxious chemical sensor, sensitive to AITC	
	• Heat sensor in Drosophila	
	• Purely chemosensory in zebrafish but sensor of warmth in medaka and arctic fish	
	• Infrared vision in snakes	
GluK2	• Role in sensing noxious cold	55 and 56
	• Initially discovered in *Caenorhabditis elegans*	
	• GluK2 mutant mice show defects in avoiding noxious cold but not cool stimuli	

### 2.2 Thermosensory Brain Regions and the Chain of Information Flow

**Fig. 1:**
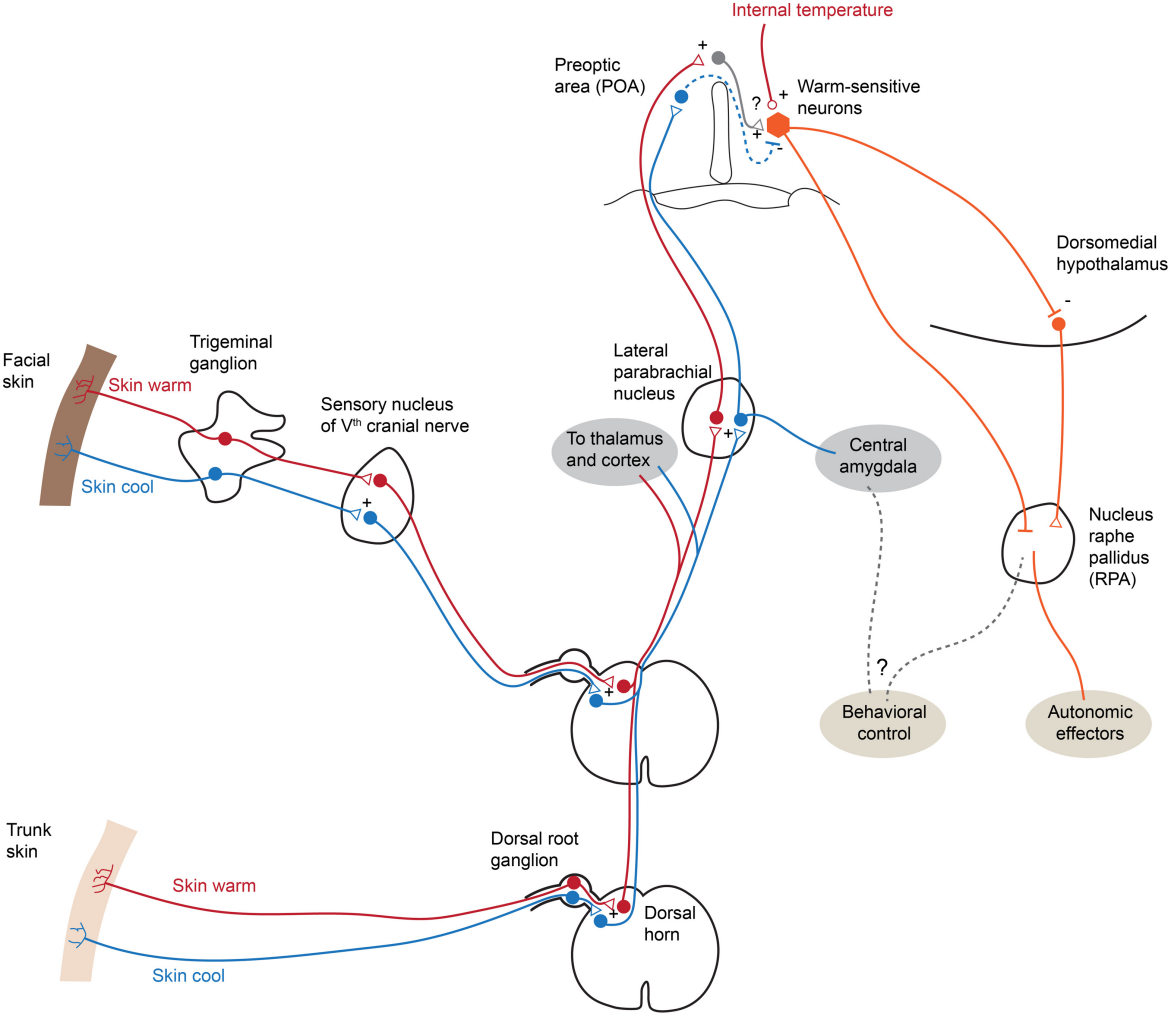
Major thermoregulatory circuits in vertebrates. This figure illustrates major pathways and “relay stations” that have been identified to carry temperature information from the skin (detected by neurons from the trigeminal or DRG) to the preoptic area, which serves as a major hub for thermoregulation. Note that some details, such as the subdivision of the parabrachial nucleus as well as information on the output pathways, have been omitted for clarity. Red lines indicate a relay of “warmth,” and blue lines a relay of “cold.” Note that the central amygdaloid nucleus has only been shown to receive information about cold. Triangles indicate likely excitatory transmission and bars likely inhibitory transmission. The question marks are meant to illustrate integrative processes that are not fully understood.

Environmental and visceral temperature sensation begins with afferent sensory neurons. Primary thermosensory afferents have cell bodies that are located within the dorsal root ganglia (DRG) or TG.^58^ These sensory afferents are pseudounipolar neurons who extend axons containing temperature-sensitive TRP channels to the surface of the skin or visceral organs.^59^ Here, these axons tile the three-dimensional space of the body and convert temperature information into membrane potential changes of the sensory neurons. These afferent sensory neurons then conduct action potentials encoding thermosensory information from the skin, past their cell body in the DRG or TG, and form glutamatergic synapses with neurons in the posterior horn of the spinal cord or the trigeminal nucleus, respectively (Fig. 1).^60^^–^^63^ The functional organization of these afferent neurons in mammals mimics the different thermal tuning profiles of TRP channels. Two classes signal noxious hot and cold stimuli, whereas another two classes are cool and warm responsive.^19^ While mammalian warm and cool responsive neurons are generally fast-adapting, this is not the case for the sensory neurons that signal noxious temperatures.^64^ This intuitively makes sense since noxious stimuli should be continuously avoided to prevent damage. Interestingly, in zebrafish, sensory neurons for innocuous thermal stimuli are non-adapting at least over timescales of tens of seconds. Instead, fast-adapting features are only computed later in the processing circuit.^65^

The processing of noxious versus innocuous temperatures already differs at the level of the spinal cord. If the environmental temperature sensed at isolated body parts is dangerous, local circuits within the gray matter of the spinal cord enact a somatic reflex to remove the affected body part as quickly as possible from the noxious temperature source (e.g., human biceps flexor withdrawal reflex when touching something hot).^66^ This circumvents higher-order processing of the signal to mitigate damage as quickly as possible. However, it should be noted that these noxious signals also get sent to higher-order processing centers, likely with the goal of both modulating current behavioral states and future behavior.^67^

The spinal afferents relay peripheral sensory signals up the ascending tract of the spinal cord to two major target areas: the thalamus^68^ from where temperature information is relayed to the cortex in mammals, and to the lateral parabrachial nucleus.^60^ Cortical encoding likely forms our conscious percept of temperature and aides in learned tasks related to thermal stimuli;^69^ however, the role of this representation in thermoregulatory behaviors is not clear given that the thalamocortical relay does not appear required for seeking out an environment with a more desirable temperature.^70^ The lateral parabrachial nucleus eventually sends the signal to subcortical forebrain structures (Fig. 1). A major pathway is to the preoptic area for integration with other relevant physiological information.^62^^,^^71^^,^^72^ However, a secondary pathway to the central amygdaloid nucleus has been implicated specifically in cold avoidance in mice, indicating a complex interplay of brain regions.^73^ Subcortical structures involved in thermoregulation are conserved across vertebrates, including zebrafish.^74^ In zebrafish, trigeminal-ganglion brainstem circuits have been implicated in thermoregulatory behaviors, and the relay of temperature information to the preoptic area as well as telencephalic structures is conserved in this species as well.^65^^,^^75^

Endothermic animals maintain a body temperature that is different from the environment and therefore monitor internal body as well as external environmental temperature.^76^^–^^78^ In mammals, POA contains warm sensitive neurons.^79^^–^^81^ These account for up to 10% of the neurons of the POA. If this area of the brain is heated in vertebrates, their bodies engage in physiological programs that lower core body temperature.^71^ There is an argument for ectothermic animals such as zebrafish not requiring internal temperature sensation since their body temperature is eventually at equilibrium with the environment. However, rapid shifts in temperature would cause a discrepancy between core and environmental temperature, even in ectotherms. This suggests that it is advantageous to have internal and external temperature sensations working in conjunction to assess the temperature state of the organism and environment. In line with this argument, there are neurons within the Drosophila brain that respond to internal temperature fluctuations mediated by the TRP channel dTRPA1.^47^ In addition, the preoptic area in sunfish was shown to contain thermosensitive neurons, indicating possible conservation of POA warm sensitive neurons across vertebrates.^82^ It is therefore likely that the sensation of internal body temperature arose independently of endothermy.

It seems that POA acts as an internal sensor specifically for brain temperature in vertebrates. It makes sense that the brain would have a dedicated set of cells to monitor the temperature of the sensitive neural tissue within the central nervous system. However, the debate around the identity of the molecular thermometer responsible for this thermosensory response is ongoing. The quest to identify the molecular sensor is complicated. Even though different TRP channels mark those POA neurons that are temperature responsive and important for the control of body temperature, it is often unclear whether these are true thermosensory neurons. Furthermore, the exact role of the TRP channels marking these neurons is often not well understood. Song et al.^45^ identified TRPM2 as a putative hypothalamic heat sensor: In cultured hypothalamic neurons, TRPM2 confers heat sensitivity, and the activity of these neurons appears to limit fever *in vivo*.^45^ However, it was later discovered that TRPM2, rather than being expressed in warm-sensitive POA neurons, indirectly activates these via a presynaptic disinhibitory mechanism, suggesting a supporting role in this process.^44^ In line with this supporting role, there is only a mild thermoregulatory phenotype when deleting TRPM2, and this sensor may rather be involved in limiting strong fever responses.^44^ Zhou et al.^39^ went a different route and created a novel POA heating system to study warm sensitive neurons *in vivo*. Combining an exploratory genetic screen with pharmacology, they could show that TRPC4 was necessary for POA heating-driven hypothermia responses.^39^ However, it is notable that the TRPC4 channel is not known to have temperature-responsive cation channel activity, and calcium imaging experiments done by the authors in human embryonic kidney cells cells also did not reveal temperature-dependent channel opening of TRPC4.^39^

Similar to the identification of molecular thermometers, the identity of “warm-sensing” or “warm-sensitive” neurons in the POA is complicated. Specifically, these neurons are often identified based on their response to warming without satisfying a clear requirement of them being thermo-sensory. Original slice work suggested that the POA indeed contains neurons that directly respond to warmth.^83^^–^^85^ However, peripheral temperature information relayed via the parabrachial nucleus results in neurons responding to peripheral warming, which have been referred to as warm-sensitive as well.^86^

To support a clearer distinction between these neuron classes, which presumably encode very different temperature information, we propose using additional criteria to better describe a neuron’s role within the thermoregulatory system. Warm/cold detecting neurons should be neurons that can be removed from their local environment and still show changes in membrane potential in response to temperature change. By this definition, the neuron must have some sort of molecular thermometer modulating membrane potential in response to temperature changes. Temperature encoding neurons on the other hand change their activity in response to temperature changes somewhere within the body but would not experience fluctuations in membrane potential associated with temperature change when isolated from the system.

A major function of the POA, aside from sensing brain temperature, is that of a major integrative center. In this role, it is thought to compute the current thermodynamic state of the system and to send signals to actuate thermoregulatory effectors to maintain temperature homeostasis.^87^^,^^88^ The preoptic area is poised for this role since it receives external and visceral temperature information from the DRG and trigeminal ganglia via the parabrachial nucleus while also being informed about brain temperature by temperature-detecting neurons within this structure.^89^ To enable regulation, there should also be an “optimal temperature” set by the POA. When this temperature is not maintained, the POA drives temperature regulation mechanisms responsible for changing temperature; be it autonomic responses such as burning brown adipose tissue (BAT), or more complex behaviors, such as making the decision to move to an environment that is closer to an ideal temperature.^65^ How this “optimal temperature” is set and encoded is still elusive, but the study of how thermoregulation is modulated by physiology may provide answers.

### 2.3 Flexibility in Thermoregulation

**Fig. 2:**
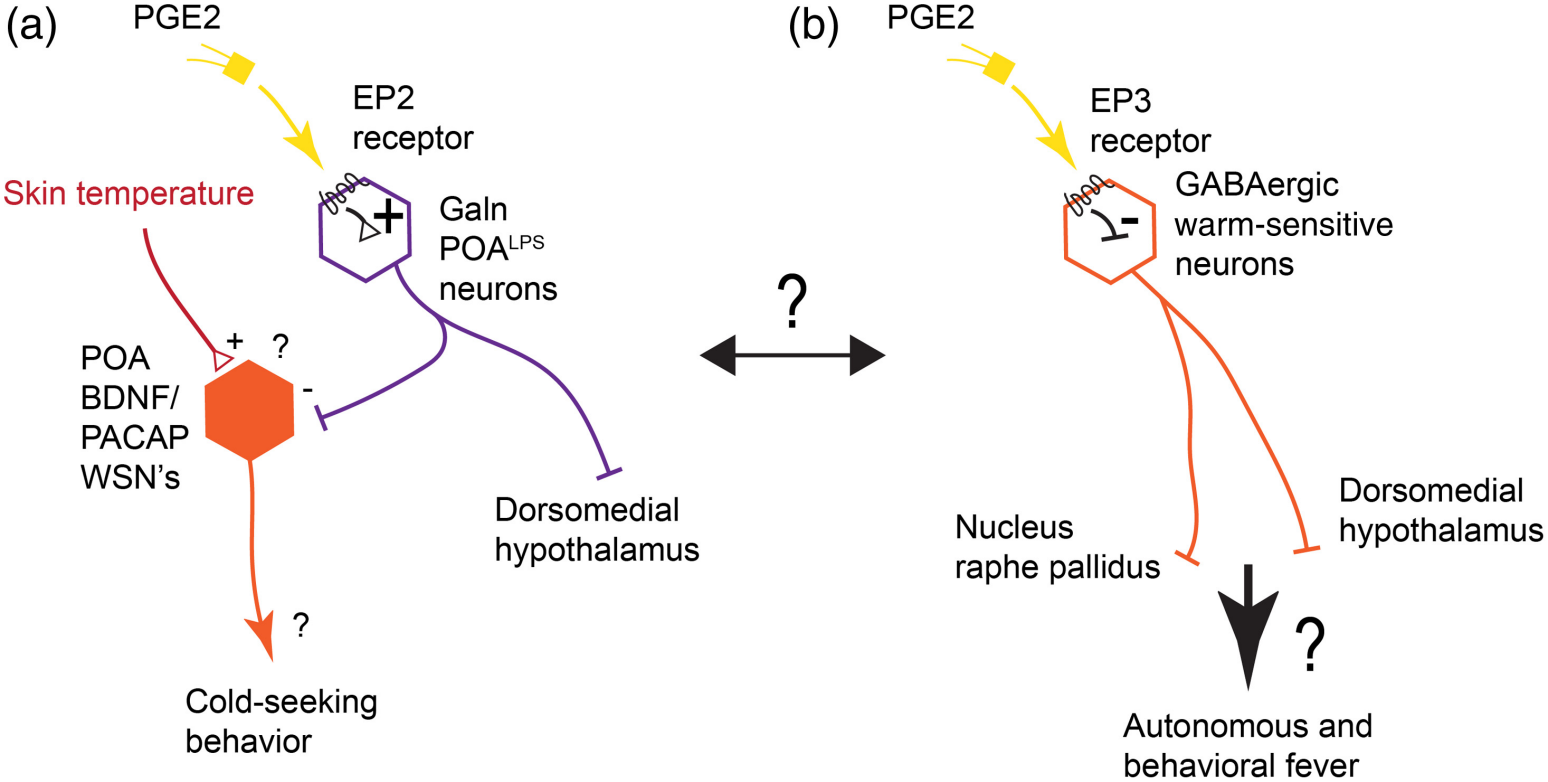
Interplay of thermal and inflammatory signals in the control of fever. (a) ${\mathrm{POA}}^{\mathrm{LPS}}$ neurons identified by Osterhout et al.^93^ increase their activity in response to prostaglandin E2 (PGE2) sensed via prostaglandin-EP2 receptors as well as in response to interleukin-1β (not shown). These neurons are Galanin-ergic and send information to the hypothalamus and provide inhibition to warm-sensitive neurons marked by BDNF/PACAP, which can induce cold-seeking behavior.^86^ This provides a possible pathway to induce warm-seeking behaviors upon induction of inflammation. (b) PGE2 sensed by EP3 receptors directly inhibits GABAergic warm-sensitive neurons in the POA, which therefore has an effect similar to cooling and induces autonomous components of fever via known pathways as well as behavioral components via unknown pathways.^94^^,^^95^ How these two pathways both established through physiological and mutagenesis studies interact is currently unknown. PGE2, prostaglandin E2; EP2, PGE2-receptor 2; EP3, PGE2-receptor 3; POA, preoptic area; BDNF, brain-derived neurotrophic factor; PACAP, pituitary adenylate-cyclase activating polypeptide; WSN, warm sensitive neuron; Galn, galanin; LPS, lipopolysaccharides.

The preoptic area is thought to act as an informational hub that amalgamates and encodes information relevant to the homeostatic state of the system for interpretation downstream. The POA receives information about internal states related to sleep, appetite, stress, and inflammation as well as various sensory information, including temperature.^90^^–^^92^ This puts the POA in a position to weigh each factor to create an assessment of the state of the system and to drive downstream processes. For example, there is a population of neurons within the preoptic area that receives inflammatory information (Fig. 2) and then alters the preferred temperature (fever) and eating behaviors when the animal is sick.^93^ How circuits within the preoptic area or possibly circuits involving other brain regions accomplish this integration of information is largely unknown.

We will focus on the complex dynamics of inflammatory fever mediated by the POA in vertebrates. We believe this to be a great example of how the POA integrates information from different systems to determine the optimal preferred temperature of the organism and direct downstream processes to reach said temperature. When vertebrates experience an infection, they raise their body temperature through autonomic and behavioral means.^89^ Ectotherms lack most of the autonomic febrile responses that endotherms have, so they exclusively rely on behavior (e.g., moving to a warmer location) to raise their body temperature in response to an inflammatory event.^96^^,^^97^

The POA receives temperature information from peripheral sensory neurons and monitors its own local temperature within the brain. In addition to encoding temperature, specific neurons within the POA are also sensitive to immunological signals.^94^ The lipid prostaglandin E2 (PGE2) is thought to be the major mediator relaying immune signals from the periphery to the POA. PGE2 is released into the brain parenchyma by endothelial cells when they detect inflammatory cytokines within the blood.^98^^–^^100^ This causes neurons within the POA, which express prostaglandin E receptors, to induce autonomic and behavioral fever responses.^101^ Research established over a decade ago strongly implicated prostaglandin receptor E3 (EP3) in the induction of fever responses.^94^^,^^102^ Fast forward to 2020 and Machado et al.^95^ published research showing that EP3 expressing preoptic area neurons are required for autonomic fever responses. Osterhout et al.^93^ on the other hand used single-cell RNA-seq and MERFISH in conjunction with patch electrophysiology and concluded that bacterial lipopolysaccharide (LPS) responsive neurons within the POA do not express EP3. Conversely, they found that prostaglandin receptor E2 (EP2) is present in these cells. Follow-up experiments confirmed that EP2 and the neurons expressing the transcript are necessary and sufficient for proper inflammatory fever responses in mice after LPS is injected.^93^ These discrepancies might hint at a complex interplay of EP2 and EP3 signaling, likely in separate populations of neurons, in the regulation of fever and sickness behaviors. However, it seems all but certain that prostaglandin E signaling induces preoptic area neurons to alter their activity, driving downstream neurogenic fever responses.

Once all the sensory signals make it to the POA and the preferred temperature is determined with respect to inflammatory states, this information is sent downstream to modulate autonomic and likely behavioral effects. A major relay downstream of the POA is the nucleus raphe pallidus (RPA).^103^ The RPA relays thermoregulatory signals to downstream autonomic control centers to maintain thermodynamic homeostasis. The processes controlled by these signals include vasodilation events that can cool an animal (e.g., vasodilation in rat tails to act as a heat sink), and BAT burning that raises the temperature of the animal.^104^ However, autonomic temperature control responses tend to be more energetically costly than behavioral means.^105^^,^^106^ There is no need to burn fat to keep yourself warm when one can find a warmer location (e.g., rats and mice will create nests) to conserve energy in the long run. So, when an endotherm becomes infected, they will still move to a warmer area instead of exclusively relying on BAT thermogenesis or muscle shivering.^105^^,^^107^ We call this behavioral drive to find warmer temperatures in the case of infection “behavioral fever” to separate it from autonomous responses.

Ectotherms must almost entirely rely on navigating the environment for their temperature control. How temperature signals are processed and relayed to drive behavioral fever is still largely unclear, but the POA was shown to be necessary for the behavioral fever response through the use of ablation techniques.^104^ However, the role of the POA in general behavioral thermoregulation is less clear, e.g., in toads lesions of the POA will disrupt LPS-induced behavioral fever but leave baseline thermoregulatory behaviors intact.^108^ In mice, LPS-responsive POA neurons can drive behavioral fever, and these neurons send projections to other regions of the brain implicated with behavioral drivers such as hunger, thirst, social interactions, and nociception.^93^ This suggests that the POA is involved in other sickness-related behaviors (e.g., less feeding, less social interactions), possibly coordinating neurogenic sickness responses across these regions. However, while this revealed an important class of neurons that when activated can alter thermoregulation by inducing behavioral fever, how this drive to warmer temperatures is actually implemented within the nervous system is still unclear.

### 2.4 Outlook: A Computational Perspective of Thermoregulation

**Fig. 3:**
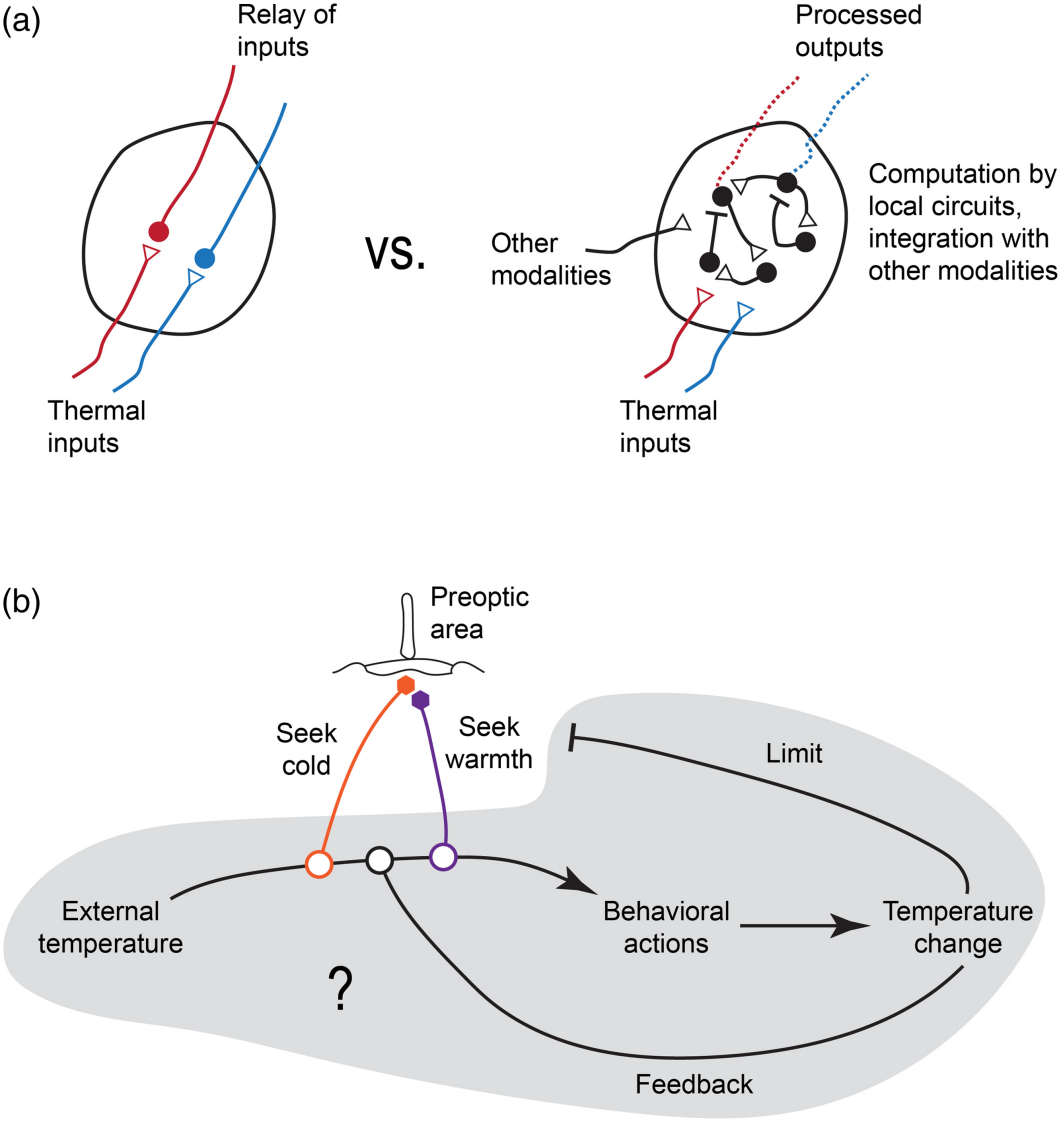
Open questions in thermoregulation. (a): Thermosensory information passes through multiple multi-sensory relay stations (also see Fig. 1). If and how local processing shapes the signals within these structures is unclear, but it is conceivable that intermediate structures not only serve as passive relays of information. (b) While neurons have been found that can induce warm- and cold-seeking behaviors, how the animal actually accomplishes these tasks is unclear. This involves appropriate processing of sensory stimuli, adjusting behaviors based on external and internal thermosensory feedback, and importantly, limiting the cold- and warm-seeking behaviors themselves to avoid entering dangerous thermal regimes.

The information presented thus far paints a picture of a basal brain structure, the POA, that acts as the temperature control module in vertebrates. Peripheral and internal thermosensors expressing TRP channels relay information about temperature to the preoptic area (Fig. 1). Here, thermosensory information is integrated with information about internal states, including inflammatory states that prominently affect the body temperature (Fig. 2). Hypothalamic output neurons then act as effectors on both autonomic and behavioral thermoregulatory responses. While this picture almost appears complete at first glance, it is largely a story of switches and relays—molecularly defined cell types that can actuate warmth seeking and sickness behaviors^86^^,^^93^ and brain regions on the input and output sides that transmit information.^71^^,^^73^^,^^109^^,^^110^ However, as in other sensory modalities such as vision and audition, it appears likely that intermediate nuclei actively process information rather than acting as simple relays [Fig. 3(a)].

Furthermore, regulation is an inherently dynamic process that heavily relies on feedback control^111^^–^^114^ and interactions across brain regions and peripheral organs.^115^ Therefore, understanding the dynamics of thermoregulation is a critical endeavor. In this last part of the review, we try to lay out possible future research avenues along these lines. These endeavors can draw on the wealth of available information on cell types, molecules, and brain regions as entry points (Fig. 3).

As outlined above, environmental temperature is sensed at the periphery by trigeminal and dorsal root ganglia neurons. This information is relayed via the brainstem to the POA and via the thalamus to the cortex.^69^^,^^104^ At the same time, the POA contains intrinsically warm-sensitive neurons that sense the internal body temperature. How these different streams of information are integrated within POA circuits and ultimately control thermoregulatory behavior is largely unclear. Lesions of the preoptic area and the thalamic relay to the cortex suggest that neither of these structures is absolutely required for thermoregulatory behaviors in vertebrates.^70^^,^^108^^,^^116^^–^^118^ However, activation of BDNF/PACAP expressing POA neurons that encode the peripheral body temperature is sufficient to induce cold-seeking behavior.^86^ This suggests that POA neurons are sufficient but may not be required to induce behavioral programs underlying thermoregulation. Rather than being involved in the underlying sensori-motor transformations from temperature to behavior, these neurons might supply an auxiliary signal that integrates information about temperature and internal states to modulate behavioral thermoregulation. Alternatively, POA neurons that encode peripheral temperature together with intrinsically warm-sensitive neurons could form a last-resort break to limit heat-seeking. This would manifest as cold-seeking upon strong activation of POA thermosensitive neurons. Together, these results suggest a tight interplay between information streams that needs untangling by observing ongoing activity across brain regions during thermoregulatory behaviors. By understanding the information flow across regions, and which regions preferentially encode which aspect of thermoregulation, sufficiency and ablation experiments can be put into a more meaningful context. For example, in larval zebrafish POA neurons encode peripheral temperature on slow timescales while major computations involved in thermoregulatory behaviors seem confined to the brainstem.^65^ However, the preoptic area has a role in reinforcing part of the thermoregulatory strategy of zebrafish, specifically reorientation maneuvers when moving away from the preference.^75^ Similar interactions might play a role in mammalian thermoregulation.

Complex coordination across brain regions is also apparent in the regulation of sickness behaviors during inflammatory responses. Neurons in both the POA and the nucleus of the solitary tract (NTS) have been shown to be responsive to LPSs and to be required and sufficient to induce sickness behaviors.^93^^,^^119^ While Ilanges et al.^119^ did not test warmth seeking and curiously observed a reduction in core temperature after LPS injection, NTS inhibition and activation do affect sickness behaviors in the same way as POA activation. This suggests a tight interplay between these brain regions, possibly with the POA serving as an integrating center and the NTS preparing commands to modulate output circuits. The involvement of the NTS is especially intriguing since it receives both somatic and autonomic information and is interconnected with the dorsal motor nucleus of the vagus.^120^ This suggests a close interplay between processes regulated by somatic motor neurons (behavior) and those regulated by autonomic motor neurons (BAT thermogenesis, vasoconstriction, and dilation). Understanding how the POA and NTS interact to process information about inflammatory states will require more than point measurements of neural activity afforded by immediate early genes and single-cell recordings. Understanding activity across these brain regions as inflammatory states develop and resolve in real time will be key to gaining insight into the modulation of thermoregulatory processes.

Another question that will benefit from a computational perspective that integrates activity information across brain regions is how “warmth-seeking” behavior is initiated during fever. Rather than being as simple as throwing a switch, behavioral strategies have to be readjusted for animals to seek out a different preferred temperature; importantly, instead of indiscriminately seeking warmth, this behavior has to be regulated to limit the temperature that is actually being sought [Fig. 3(b)]. Notably, this behavioral change could arise due to two broad classes of effects: The sensation of temperature could simply be altered—the thermal preference changes because at the sensory level 30°C now feels like 29°C; alternatively, a sense of absolute temperature is maintained, but the transformation of thermosensory information to thermoregulatory behaviors is altered. There are indications that sympathetic stimulation can influence the response of trigeminal cold-responsive neurons,^121^ and prostaglandin E2, a major mediator of fever, can directly sensitize the capsaicin response of TRPV1 channels in the lung.^122^ However, this latter effect should rather reduce preferred temperatures as it would increase the feeling of warmth. It is therefore unclear whether the behavioral expression of fever is caused by a coordinated change in thermal sensitivity at the periphery. A perhaps more likely scenario is modulation of processing at points of integration. And indeed, inhibitory POA neurons that increase their activity upon inflammatory stimulation appear to target BDNF/PACAP expressing POA neurons^93^ that have been shown to drive cold-seeking upon exogenous activation.^86^This suggests the presence of a subtractive signal that can modulate thermoregulatory behaviors. As noted above, since the POA itself may be dispensable for thermal navigation, this signal might serve more as a source of modulation for circuits that process thermosensory stimuli to enact the seeking of preferred temperatures. Alternatively, the POA may signal information about the preferred temperature itself, and departures from this temperature are used by downstream circuits to guide behaviors. This would be a central implementation of a sensory motif that has been discovered in Drosophila larvae where symmetric hot and cold avoidance are generated by switching inputs from hot and cold thermosensors around the preferred temperature.^123^

### 2.5 Toolkit for Future Thermoregulation Research

A broad set of molecular, genetic, and physiological tools, from cell-type specific transgenes, single-cell RNA sequencing to ablations, inactivations, and electrophysiology has identified key players in vertebrate thermoregulatory pathways. Ground-breaking behavioral and imaging work in invertebrate models has uncovered important principles of thermosensory encoding and processing,^8^^,^^124^^–^^128^ whereas imaging in the mouse spinal cord has revealed a high complexity of thermosensory representation already at the initial stages of processing.^129^ We believe that larval zebrafish can serve as a vertebrate model of intermediate complexity to uncover important computational principles of thermoregulation.

The large array of subcortical brain regions implicated in thermoregulation and its modulation in mammals is conserved in zebrafish^74^ and brain regions such as the POA or nucleus RPA contain expected temperature-encoding neurons in zebrafish.^65^^,^^130^ With its amenability to whole-brain imaging in restrained^65^^,^^131^^–^^133^ and freely swimming conditions,^134^^,^^135^ larval zebrafish specifically allows to observe the relationship of neural activity across multiple brain regions during behavioral thermoregulation. This is critical for identifying computational principles that allow brains to “implement a thermostat.” Modeling work in larval zebrafish led to an initial quantitative description of heat avoidance behavior that predicted the role of temperature changes in controlling thermal navigation.^136^^,^^137^ Brain-wide imaging studies on the other hand identified neurons encoding temperature, temperature change, and neurons that seem to integrate over successive temperature fluctuations.^65^^,^^75^^,^^138^ Interestingly, these are signals that are important for engineered feedback controllers, including temperature control systems.^139^ Paired with deep analysis,^138^^,^^140^^,^^141^ larval zebrafish could therefore yield important insight into how activity across brain regions orchestrates thermoregulation. Indeed, observation of neural activity across brain regions led to the first realistic circuit model of heat avoidance,^65^ illustrating the power of this approach. While this model is incomplete, lacking modulatory inputs and information on the processing of cold stimuli, it provides a demonstration of how computational models could be constrained by large-scale functional imaging data obtained during a thermoregulatory task. Notably, ongoing improvements in voltage imaging^142^^–^^144^ promise to greatly improve the utility of circuit models in the future. The observation of accurate spike timing and possibly sub-threshold events across brain regions will greatly enhance our understanding of computational principles.

The combination of imaging and modeling will complement ongoing research on thermoregulation across species, putting identified cell types and their role into the context of ongoing sensation and behavior. Modeling will also be instrumental in understanding the role of the cortical representation of temperature signals in mammals^69^ and how cortical processing shapes and integrates these signals with other modalities. This work would likely draw on a combination of advances in wide-field and 2-photon imaging as well as neuropixel recordings.

## 3 Conclusion

Research on thermoregulation has uncovered key players and principles, from gene families encoding thermoreceptors via input and output pathways of the thermoregulatory and thermosensory system all the way to ideas of how thermosensory information is integrated with internal states to adjust thermoregulation to the need of animals. However, many interesting questions remain. There seem to be discrepancies between the properties of TRP channels *in vitro* and *in vivo* such as the apparent disagreement of thermal thresholds of TRPV1 in cell culture versus their role in the mouse TG. These need to be resolved to fully understand the molecular code of thermosensation. The molecular thermosensor of intrinsically warm-detecting neurons in the POA is still unknown. And studies of the computational interplay that links thermosensation to thermoregulatory behavior are still in their infancy, and we understand very little about how putative circuits that control thermoregulatory behaviors are influenced by inflammatory signals originating in the POA or NTS. Addressing such computational aspects of thermoregulation in simpler models can reveal worthwhile entry points of study in organisms in which access to the brain is more limited.

## Data Availability

No code or data were generated for this review article.
